# A single subcutaneous dose of eprinomectin (Eprecis^®^) is effective against common gastrointestinal nematodes and lungworms in experimentally infected lactating goats

**DOI:** 10.1186/s13071-024-06301-w

**Published:** 2024-05-10

**Authors:** Alexandra Beck, Sarah Thomson, David Reddick, Rike Brunner, Dana Campbell-Wilson, Damien Achard, Naomi Isaka, Anne Trotel, Hamadi Karembe

**Affiliations:** 1 Moredun Scientific, Pentlands Science Park (PSP), Bush Loan, Penicuik, Midlothian EH26 0PZ UK; 2 Argenta DE Geyersperger Str. 27, 80689 München, Germany; 3 Dana Campbell Consultants Ltd, 15 Justice Park, Oxton, Lauderdale TD2 6NZ UK; 4 Ceva Santé Animale, 10 Avenue de La Ballastière, 33500, Libourne, France

**Keywords:** Gastrointestinal nematodes, Lungworms, *Teladorsagia circumcincta*, *Haemonchus contortus*, *Trichostrongylus colubriformis*, *Dictyocaulus filaria*, Lactating goats, Controlled trial, Treatment, Eprinomectin

## Abstract

**Background:**

The health and productivity of dairy goats continue to be impacted by gastrointestinal nematodes (GIN) and lungworms (LW). Eprinomectin (EPN) is frequently selected for treatment because it is generally effective and does not require a milk withdrawal period. However, some factors, such as lactation, can have an impact on EPN pharmacokinetics and potentially its efficacy. To evaluate whether this can alter the efficacy of Eprecis^®^ 2%, an eprinomectin injectable solution, a study was performed in lactating goats using the dose currently registered in cattle, sheep and goats (0.2 mg/kg).

**Methods:**

This study was a blinded, randomized, controlled trial performed according to the VICH guidelines. Eighteen (18) worm-free lactating goats were included and experimentally challenged on day 28 with a mixed culture of infective gastrointestinal and lung nematode larvae (*Haemonchus contortus, Trichostrongylus colubriformis, Teladorsagia circumcincta, Dictyocaulus filaria*). At D-1, fecal samples were collected to confirm patent infection in all animals. On D0, the goats were randomly allocated into two groups of nine goats; group 1 was treated with Eprecis^®^ 2% at 0.2 mg/kg BW by subcutaneous injection, while group 2 remained untreated. Fecal samples for egg counts were collected from all animals on days 3, 5, 7, 9, 11 and 14. On D14, all goats were killed, and the abomasum, small intestine and lungs were removed, processed and subsampled to record the number and species of worms.

**Results:**

The treatment was well tolerated. After treatment, the arithmetic mean FEC decreased in the treated group and remained < 5 EPG until the end of the study, while the arithmetic mean FEC in the control group remained > 849.0 EPG. At D14, goats in the treated group had very limited or zero total worm counts, whereas all animals from the control group had a high worm burden. The measured efficacy was 100.0% against *H. contortus* and *T. colubriformis,* 99.9% against *T. circumcincta* and 98.0% against *D. filaria*.

**Conclusions:**

Eprinomectin (Eprecis^®^, 20 mg/ml), administered at the label dose (0.2 mg/kg), is highly effective against gastrointestinal nematodes and lungworms in lactating goats.

**Graphical abstract:**

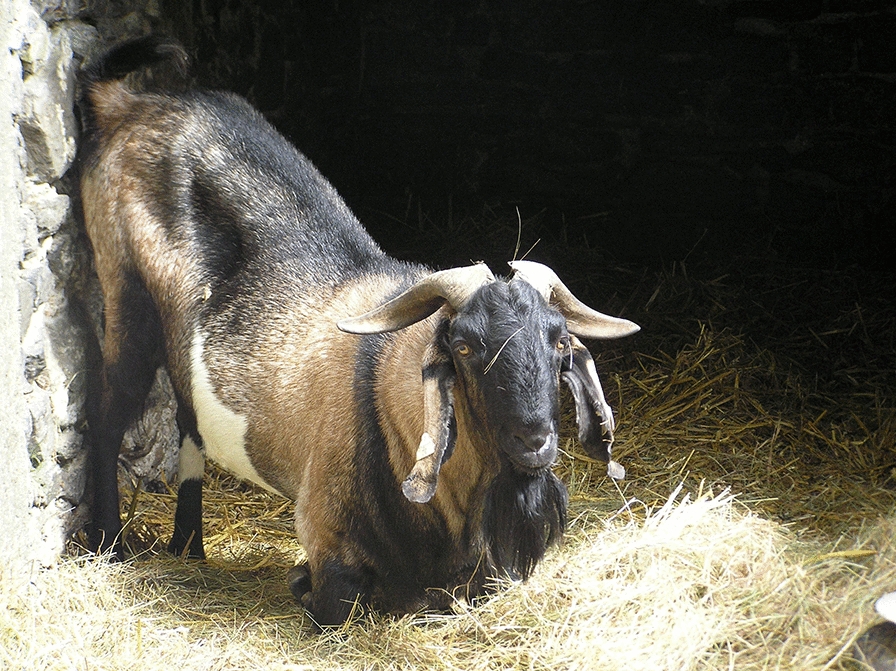

**Supplementary Information:**

The online version contains supplementary material available at 10.1186/s13071-024-06301-w.

## Background

Worm infections, including infections with gastrointestinal nematodes (GIN) such as *Teladorsagia* spp., *Trichostrongylus* spp. and *Haemonchus* spp. and lungworms (LW) such as *Dictyocaulus* spp., are common in goats worldwide. These nematode infections are still major financial issues due to both production losses and animal health and welfare. The control of GIN or LW infections in goats relies on pasture management, the development of host immunity against these parasites and the prudent use of a suitable anthelmintic. Indeed, in the context of increasing levels of resistance to anthelminthics [1] and growing concerns regarding the environmental impact of anthelminthics residues in dung [2, 3], it is important to implement a tailored strategy for parasitism management at the farm level that includes the rationalized use of anthelmintics.

In dairy goats, eprinomectin (EPN) is frequently selected for treatment purposes because there is no milk withdrawal period. EPN is available for goats in two formulations: a pour-on (e.g. Eprinex Multi^®^, Boehringer Ingelheim) and an injectable formulation (Eprecis^®^ 20 mg/ml solution for injection, Ceva Santé Animale) [4]. Pour-on administration of eprinomectin is known to result in highly variable exposure to the drug among individuals, as measured by the area under the curve (AUC). The AUC is considered a good predictor of the anthelmintic efficacy of macrocyclic lactones [5]. Another factor to consider in dairy goats is lactation. The change in adipose tissue during this period has been hypothesized to have an impact on the bioavailability of anthelminthic molecules. Indeed, a reduced bioavailability has been reported for topical EPN in lactating goats compared to dry goats [6]. This finding, along with variable bioavailability between individuals, is believed to play a role in reported cases of loss of efficacy or resistance following EPN pour-on application in goat herds [7, 8].

In goats, the injectable formulation of EPN is known to result in 2.5 times greater bioavailability compared to topical administration [9], but until now, published evidence of its efficacy in lactating goats was lacking, although studies have reported that injectable EPN is associated with high efficacy in lactating sheep [10, 11] and nonlactating goats [12]. To determine whether lactation could alter the efficacy of injectable EPN, this study was conducted in lactating goats at the recommended label dose for cattle, sheep and goats (0.2 mg/kg bodyweight).

### Methods

### Ethical approval

This research was implemented under experimental conditions by Moredun Scientifics at the Pentlands Science Parks Facilities (UK). The protocol was reviewed by the Moredun Animal Welfare and Ethical Review Board, and the study was performed under the UK Home Office Project License (no. PFA7E7AD6) following the Animal Scientific Procedures Act 1986.

### Study design

This study was a blinded, randomized, controlled and VICH GCP-compliant study conducted according to VICH GL 7 (Efficacy of Anthelmintics) and VICH GL14 (Anthelmintics: Caprine).

### Animal characteristics at selection

Eighteen crossbred goats (Saanen/Toggenburg/Alpine/Nubian) from a conventional dairy goat farm were selected and included. They were raised indoors with no prior anthelmintic treatment. Each animal was identified using ear tags. All goats were primiparous and aged > 1 year at the start of the study. The lactation stage at D0 was approximately 3 months postpartum.

### Experimental infection and randomization

An acclimatization period of 16 days prior to parasitological challenge (on D-28) was applied, and all the subjects were housed in one group (prior to treatment on D0) in a large standard barn accommodation at Moredun’s Pentlands Science Park on straw. The worm-free status was checked by performing individual fecal egg counts on D-43, D-31, D-30 and D-28. A clinical examination was performed by a veterinarian on the day of challenge (D-28) to confirm good general health and suitability for the inclusion of animals. The same day, a mixed culture of infective larvae of *Haemonchus contortus* (field isolate from Scotland), *Trichostrongylus colubriformis* (laboratory isolate) and *Teladorsagia circumcincta* (field isolate from Scotland) and a culture of larvae of *Dictyocaulus filaria* (field isolate from England) were administered per os (p.o.) (Table 1). The average numbers complied with the recommended numbers for parasite infection models for anthelminthic evaluation in goats (according to VICH GL14). The isolates used in the study were recent European strains isolated from the field (except for *T. colubriformis* from a laboratory isolate). A single dose of GIN and a separate suspension of *D. filaria* were administered orally using a syringe (10 ml each).

**Table 1:** Composition of the challenge dose and recommendations

Species	Approximate number of larvae per challenge dose in the study	Recommended numbers of larvae to produce adequate infections (VICH GL14)
*Haemonchus contortus*	4002	400–4000
*Trichostrongylus colubriformis*	5920	3000–6000
*Teladorsagia circumcincta*	9985	6000–10,000
*Dictyocaulus filaria*	1065	1000–2000

One day prior to treatment (D-1), individual fecal egg counts were performed to monitor egg shedding (ranging from 108 to 3285 EPG), reflecting a patent infection in individual animals. Altogether, 18 animals were enrolled in the study and randomized based on their D-1 fecal egg counts according to a randomization list with a block size of two, animals being ranked from largest to smallest fecal egg count. Within each set of two, one animal was randomly allocated to each group. Only the dispenser and the product administrator were aware of the allocation of animals to their groups, and they were not involved in the observation of the animals.

### Animal husbandry

On D0, the goats were allocated into two groups of nine animals and housed separately from that time. Animals were provided with a minimum of 1.3 m^2^ of floor space per animal and bedded on straw in a deep litter system. The accommodation was held at ambient temperature and had natural lighting and ventilation, with artificial lighting available if needed. The goats were provided a commercially available balanced concentrate ration at an appropriate amount for their age and size daily, and hay was provided ad libitum. Water was available ad libitum. No routine treatment occurred during the study period. Animals were milked once a day during the study, from arrival until D13. Individual milk production was not recorded during the study, but the total milk yield of all animals was recorded. The general health of the animals was assessed twice daily from arrival until the end of the study. Any sign of abnormal behavior or a change in feeding habits was reported. Fecal egg shedding was regularly monitored on D0, D3, D5, D7, D9, D11 and D14.

### Treatment and follow-up

On D0, an individual weighing was performed to calculate the suitable treatment dose for each goat. The nine animals in the treated group received the dose recommended in the Summary of Product Characteristics (SPC): 0.1 ml/10 kg body weight of Eprecis^®^ 20 mg/ml (Ceva Santé Animale) by subcutaneous injection into the left side of the neck. When the required dose volume exceeded 0.6 ml (for goats weighing > 60 kg), the dose was split into two injection sites on the left and right necks. All animals from the control group remained untreated.

At the end of follow-up, on D14, the goats were weighed and then humanely killed to allow gastrointestinal tract and lungs sampling for parasite counts. The abomasum, small intestine and lungs were removed. The digestive organs were opened, and the mucosal surface was washed with warm physiological saline (0.9% NaCl), placed into incubators, rinsed and subsampled. The lungs were opened and put into a saline solution. Then, the *D. filaria* were removed, placed into subsample pots with saline and incubated. The samples were then cut into smaller sections, washed again with saline and then sieved to collect larvae and worms. The number and species of each target worm were recorded, as well as the sex (male or female) of all worms after examination under a microscope. Male subjects were used for parasite species identification.

### Efficacy assessment and statistical analysis

Descriptive statistics, including minimum and maximum values, medians, arithmetic means (A Mean) and geometric means (G Mean) and their standard deviations were calculated for the fecal egg and worm counts.

To assess the effect of Eprecis^®^, the nematode counts of treated animals were compared to those of control animals using Wilcoxon tests, with significance set at the 0.05 level.

The effectiveness of Eprecis^®^ against each worm species was calculated as the difference in geometric means of the counts between the control group and the treated group at D14, expressed as a percentage based on the geometric mean of the control group counts. To allow for zero counts, geometric means were computed on the log scale using log(count + 1), as per VICH GL7, and 1 was subtracted from the final result.

Statistical analyses were performed using SAS version 9.4 software.

## Results

### Analyzed population

The characteristics of all included animals (*N* = 18) are presented in Table 2. Their ages ranged from 15 to 25 months, and their body weight at D0 was between 40.8 kg and 75.2 kg. Only one goat was above 60 kg and required two different injection sites (0.40 and 0.35 ml). The lactation stage at D0 was ranging from 3 months 14 days to 4 months 12 days post-partum.

**Table 2:** Characteristics of the included animals (*N* = 18)

Goat ID	Group*	Age on D0	Months postparturition on D0	Body weight on D0	Fecal egg counts on D-1
56	1	1 years 8 months 11 days	3 months 14 days	48.0	954
5339	1	1 years 3 months 4 days	4 months 7 days	46.4	1170
58	1	1 years 8 months 9 days	4 months 12 days	45.6	140
62	1	2 years 1 months 8 days	4 months 10 days	48.4	2115
5288	1	2 years 0 months 21 days	4 months 7 days	53.4	283
64	1	1 years 4 months 9 days	3 months 15 days	40.8	141
5293	1	2 years 0 months 19 days	4 months 3 days	59.4	783
59	1	1 years 8 months 9 days	4 months 11 days	75.2	1575
5308	1	2 years 0 months 13 days	4 months	47.4	1251
57	2	2 years 0 months 9 days	3 months 14 days	46.4	924
63	2	2 years 1 months 8 days	4 months 12 days	54.2	2043
53	2	1 years 8 months 29 days	4 months 9 days	58.8	1002
61	2	1 years 8 months 9 days	4 months 4 days	43.6	381
5233	2	2 years 1 months 21 days	4 months 3 days	63.4	1233
41	2	1 years 4 months 16 days	4 months 12 days	57.8	282
5264	2	2 years 1 months 15 days	3 months 28 days	53.2	705
60	2	1 years 8 months 14 days	3 months 14 days	46.2	108
4519	2	1 years 9 months 13 days	3 months 28 days	60.2	3285

^*^Group 1 = treated; Group 2 = control

### Fecal egg count evolution

Animals in both groups were negative prior to challenge (FEC = 0). Following the challenge, the fecal egg counts in all animals increased, reaching similar mean counts in both groups. After treatment, the mean fecal egg counts decreased and remained < 2.4 EPG (A Mean) and at 1.4 EPG (G Mean) until the end of the study, while they remained > 849.0 EPG (A Mean) and 562.5 EPG (G Mean) in the control animals throughout the study (Table 3 and Additional file 1: Fig. S1).

**Table 3:** FEC changes in the control and treated groups (*N* = 18)

Day	Statistics	Treated (*N* = 9)	Control (*N* = 9)	*p*-value
− 43 to -28	Median G Mean	0.0 0	0.0 0	
− 1	Median G Mean	954.0 649.4	924.0 735.8	1.0000
3	Median G Mean	0.0 0.2	1041.0 1059.9	0.0003
5	Median G Mean	1.0 1.3	1206.0 1147.8	0.0004
7	Median G Mean	0.0 0.7	1008.0 1005.9	0.0004
9	Median G Mean	1.0 1.0	975.0 925.4	0.0004
11	Median G Mean	0.0 0.6	498.0 579.0	0.0003
14	Median G Mean	0.0 0.7	402.0 584.5	0.0004

### Nematode counts

The total numbers of adult worms recovered on D14 in the sampled organs (abomasum, small intestine and lung) are summarized in Table 4. The arithmetic and geometric means (and their standard deviations) were calculated, and both showed the same significant differences: in the treated group, the geometric means were null (small intestine and lung) or very low (abomasum), while they were high in the control group, particularly in the digestive organs (G Means of 1785.1 in the abomasum and 2094.3 in the small intestine) compared to the lung (G Mean = 10.3).

**Table 4:** Adult worm counts in predilection sites at D14 (*N* = 18)

Group	Statistics	Abomasum *(Haemonchus contortus and Teladorsagia circumcincta)*	Small intestine *(Tolubriformis colubriformis)*	Lung *(Dictyocaulus filaria)*
Treated (*N* = 9)	A Mean (St Dev)	22.2 (26.4)	0.00 (0.0)	0.3 (1.0)
	G Mean p-value	4.7 0.0003	0.0 0.0002	0.2 0.0010
Control (*N* = 9)	A Mean (St Dev)	3205.6 (2704.6)	2322.2 (922.3)	24.8 (25.1)
	G mean	1709.7	2131.3	13.0

**Fig. 1:**
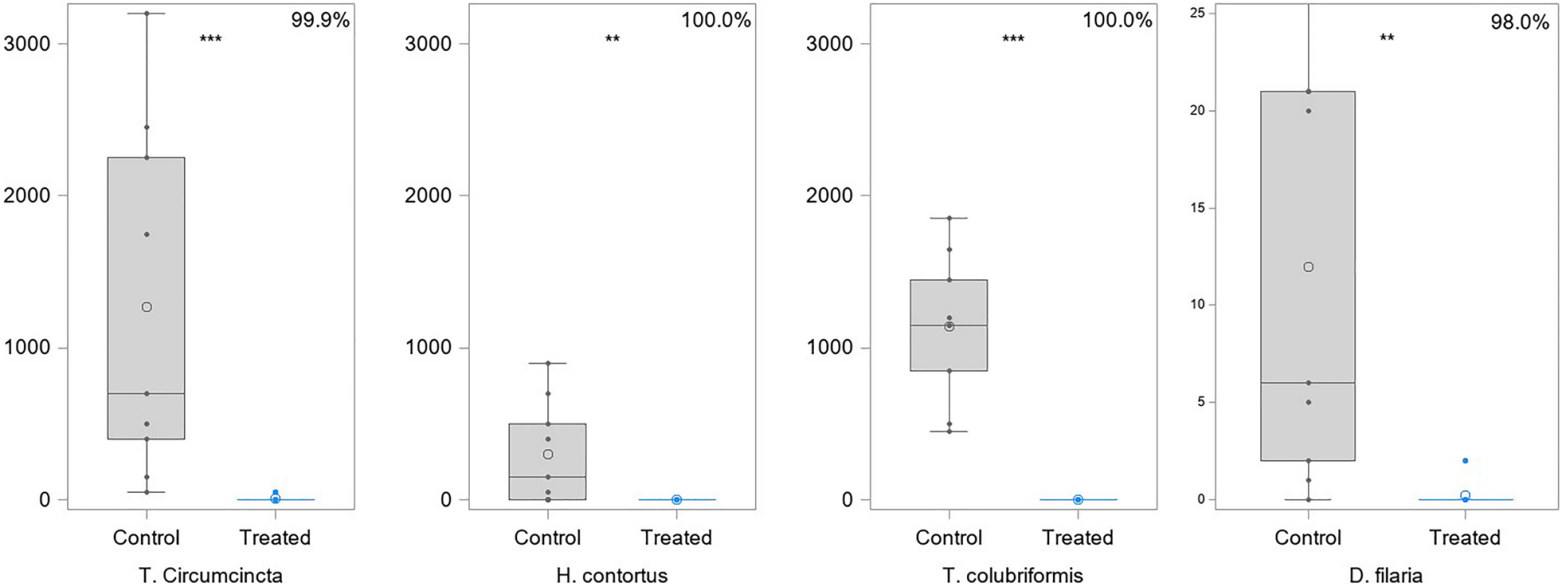
Nematode counts in the control and treated groups

The distributions of adult worm counts by nematode species are presented in Table 5 and Fig. 1. The percentage of efficacy calculated using both calculated means provided similar results, with 99.9% efficacy against *T. circumcincta*, 100.0% efficacy against *H. contortus* and *T. colubriformis* and 98.0% efficacy against *D. filaria.* These results are greater than the minimum required 90% effectiveness (VICH GL14). All nematode species were found at necropsy in almost all animals from the control group (Additional file 2: Table S2). Indeed, at least six goats from the control group were considered infected for each nematode species. However, regarding *H. contortus*, only 50 parasites could be collected at necropsy in one goat and none in two others. Regarding *D.filaria*, no parasite could be isolated in one goat. In the treated group, almost all goats had negative counts for all four nematode species, except for one goat with some *T. circumcincta* and another goat with some *D. filaria*.

**Table 5:** Adult worm counts at D14 (*N* = 18)

Worm species	Statistics	Treated (*N* = 9)	Control (*N* = 9)	*p*-value (Wilcoxon test)
*Teladorsagia circumcincta*	Median G Mean Min–max Efficacy (%)	0.0 0.0 0.0; 50.0 99.6	700.0 684.8 50.0; 3200.0	0.0003
*Haemonchus contortus*	Median G Mean Min–max Efficacy (%)	0.0 0.0 0.0; 0.0 100.0	150.0 45.3 0.0; 900.0	0.0053
*Trichostrongylus colubriformis*	Median G Mean Min–max Efficacy (%)	0.0 0.0 0.0; 0.0 100.0	1150.0 1033.5 450.0; 1850.0	0.0002
*Dictyocaulus filaria*	Median G Mean Min–max Efficacy (%)	0.0 0.1 0.0; 2.0 98.1	6.0 6.6 0.0; 32.0	0.0016

Wilcoxon tests calculated on the mean values of worm counts showed significant differences between groups for *D. filaria* (*P* = 0.0072), *H. contortus* (*P* = 0.0074), *T. circumcincta* (*P* = 0.0012) and *T. colubriformis* (*P* = 0.0008) (Table 5).

Post hoc Fisher’s exact tests also showed that for all species there was a significant difference in the proportion of zero counts between the treatment and control groups, indicating that the treatment was effective at eliminating these four worm species on day 14 (*P* = 0.0034 for *D. filaria*, *P* = 0.0090 for *H. contortus*, *P* = 0.0004 for *T. circumcincta* and *P* = 0.0000 for *T. colubriformis*).

### Changes in health status and body weight

Overall, the treatment was well tolerated.

Body weights were recorded on D0 and D14 just before necropsy. The mean body weights in both groups increased slightly from D0 to D14 (by + 2.8 kg in the treated group and + 1.6 kg in the control group).

## Discussion

In this study, we reported an anthelmintic efficacy ≥ 98.0% in lactating goats following experimental challenge with *H. contortus, T. colubriformis, T. circumcincta and D. filaria* and subsequent treatment with a single subcutaneous injection of 0.2 mg/kg eprinomectin, the registered dose for cattle, sheep and goats. The same levels of efficacy were reported in naturally infected lactating sheep [10] and in nonlactating goats artificially challenged [12] with *H. contortus* (efficacy = 99.8% and 97.8%, respectively, in both studies) and *T. colubriformis* (efficacy = 99.7% and 98.7%, respectively) using the same eprinomectin injectable formulation and the same dose (0.2 mg/kg). Taken together, these findings suggest that lactation does not have a decisive impact on the efficacy of eprinomectin against internal parasites in goats when given subcutaneously and that the dose currently registered for eprinomectin injection in small ruminants is effective in both lactating and nonlactating goats.

Eprinomectin is the last macrocyclic lactone to be developed and used in food-producing animals. It is highly potent against gastrointestinal parasites, lungworms and several ectoparasites. Eprinomectin has a low milk partition, making it a convenient product for the control of parasites during lactation. Until recently, eprinomectin was only available as a pour-on formulation for cattle, but it was quickly used off-label at half the current registered dose in small ruminants (sheep and goats), with oral application being frequently reported [13]. Eprinomectin was subsequently approved for use in sheep and goats in pour-on and injectable formulations at 1 mg/kg and 0.2 mg/kg, respectively. A lower and variable systemic bioavailability of eprinomectin was reported in goats following topical application [14]. Lactation also has a significant effect, as its bioavailability is lower in lactating goats than in nonlactating goats [6]. Consequently, topical dose rates > 0.5 mg/kg (the cattle dose) are required for significant anthelmintic efficacy in goats [15], and higher dose rates may be considered as required when used during lactation. The results of this study confirm that no increase in the dose administered is required when using this injectable formulation in lactating goats.

## Conclusions

This study demonstrated the effectiveness of 0.2 mg/kg BW eprinomectin (Eprecis^®^ 20 mg/ml Solution for injection), a dose already registered in cattle, against *H. contortus, T. colubriformis, T. circumcincta* and *D. filaria* in lactating goats. The subcutaneous administration of a single dose significantly reduced the worm counts of common gastrointestinal and pulmonary worms in goats. In the future, a similar study design may be replicated to assess the impact of an antiparasitic treatment on milk production, overall zootechnical performance during lactation and, ultimately, financial consequences for breeders.

### Supplementary Information

**Additional file 1: Figure S1.** Individual evolutions of FEC from D-1 prior to treatment to D14 (N = 18).

**Additional file 2: Table S2.** Individual nematode counts at necropsy (N = 18).

## Data Availability

Data supporting the conclusions of this article are included within the article and its additional files. Further data of interest will be available from the corresponding author upon request.

## Abbreviations

A Mean: Arithmetic mean
AUC: Area under the curve
BW: Body weight
D: Day
EPG: Eggs per gram
EPN: Eprinomectin
GIN: Gastrointestinal nematode
G Mean: Geometric mean
kg: Kilogram
LW: Lungworm
mg: Milligram
mL: Milliliter
N: Number
St Dev: Standard deviation

## References

[1] Vineer HR, Morgan ER, Hertzberg H, Bartley DJ, Bosco A, Charlier J, Chartier C, Claerebout E, De Waal T, Hendrickx G, Hinney B (2020). Increasing importance of anthelmintic resistance in European livestock: creation and meta-analysis of an open database. Parasite.

[2] Beynon SA (2012). Potential environmental consequences of administration of anthelmintics to sheep. Vet Parasitol.

[3] Lumaret JP, Errouissi F (2002). Use of anthelmintics in herbivores and evaluation of risks for the nontarget fauna of pastures. Vet Res.

[4] Veterinary Medicines Directorate, UK. Summary of product characteristics, revised in September 2022. https://www.vmd.defra.gov.uk/productinformationdatabase/files/SPC_Documents/SPC_793085.pdf. Accessed 02 Nov 2023.

[5] Rostang A, Devos J, Chartier C (2020). Review of the Eprinomectin effective doses required for dairy goats: where do we go from here?. Vet Parasitol.

[6] Dupuy J, Chartier C, Sutra JF, Alvinerie M (2001). Eprinomectin in dairy goats: dose influence on plasma levels and excretion in milk. Parasitol Res.

[7] Murri S, Knubben-Schweizer G, Torgerson P, Hertzberg H (2014). Frequency of eprinomectin resistance in gastrointestinal nematodes of goats in canton Berne. Switzerland Vet Parasitol.

[8] Couasnon F. Méthode de détection des suspicions d’inefficacité de l’éprinomectine administrée par voie topique chez les caprins laitiers. Thèse d’exercice vétérinaire, Ecole vétérinaire de Nantes Oniris. 2019. https://books.google.fr/books?id=2yNMzQEACAAJ.

[9] Lespine A, Sutra JF, Dupuy J, Alvinerie M (2003). Eprinomectin in goat: assessment of subcutaneous administration. Parasitol Res.

[10] Termatzidou SA, Arsenopoulos K, Siachos N, Kazana P, Papadopoulos E, Achard D, Karembe H, Bramis G, Arsenos G (2019). Anthelmintic activity of injectable eprinomectin (Eprecis^®^ 20 mg/ml) in naturally infected dairy sheep. Vet Parasitol.

[11] Termatzidou SA, Siachos N, Kazana P, Sotiraki S, Saratsi K, Achard D, Karembe H, Bramis G, Kanoulas V, Arsenos G (2020). Effect of injectable eprinomectin on milk quality and yield of dairy ewes naturally infected with gastrointestinal nematodes. Vet Parasitol.

[12] Brique-Pellet C, Ravinet N, Quenet Y, Alvinerie M, Chartier C (2017). Pharmacokinetics and anthelmintic efficacy of injectable eprinomectin in goats. Vet Parasitol.

[13] Badie C, Lespine A, Devos J, Sutra JF, Chartier C (2015). Kinetics and anthelmintic efficacy of topical eprinomectin when given orally to goats. Vet Parasitol.

[14] Alvinerie M, Lacoste E, Sutra JF, Chartier C (1999). Some pharmacokinetic parameters of eprinomectin in goats following pour-on administration. Vet Res Commun.

[15] Chartier C, Etter E, Pors I, Alvinerie M (1999). Activity of eprinomectin in goats against experimental infections with *Haemonchus contortus, Teladorsagia circumcincta* and *Trichostrongylus colubriformis*. Vet Rec.

