# Supplementary figures and images for: A single subcutaneous dose of eprinomectin (Eprecis®) is effective against common gastrointestinal nematodes and lungworms in experimentally infected lactating goats
Source: Parasit Vectors. 2024 May 10;17:211. doi: 10.1186/s13071-024-06301-w (PMC11084049; doi:10.1186/s13071-024-06301-w)

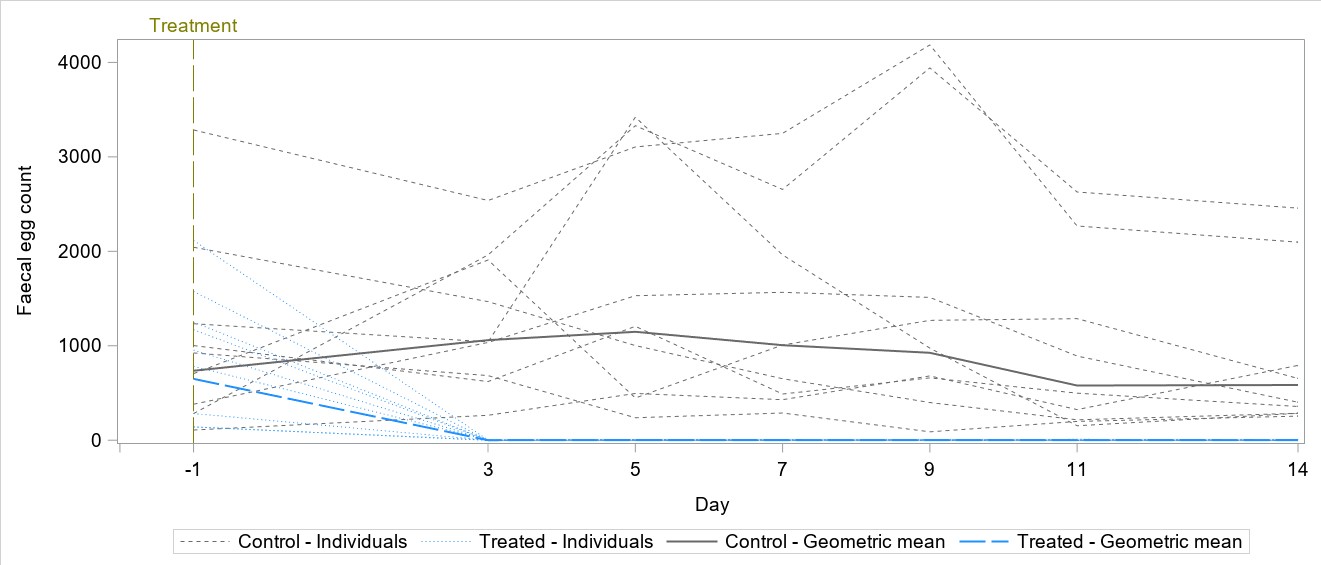

Supplement: Supplementary file 1 — Additional file 1: Figure S1. Individual evolutions of FEC from D-1 prior to treatment to D14 (N = 18). [file 13071_2024_6301_MOESM1_ESM.jpg]
