# Supplementary material for: A single subcutaneous dose of eprinomectin (Eprecis®) is effective against common gastrointestinal nematodes and lungworms in experimentally infected lactating goats
Source: Parasit Vectors. 2024 May 10;17:211. doi: 10.1186/s13071-024-06301-w (PMC11084049; doi:10.1186/s13071-024-06301-w)
Supplement: Supplementary file 2 — Additional file 2: Table S2. Individual nematode counts at necropsy (N = 18). [file 13071_2024_6301_MOESM2_ESM.docx]

**Additional file 2: Table S2: Individual nematode counts at necropsy (N = 18)**

| **Goat ID** | **Group*** | ***T. circumcincta*** | ***H. contortus*** | ***T. colubriformis*** | ***D. filaria*** |
| --- | --- | --- | --- | --- | --- |
| 5308 | 1 | 0 | 0 | 0 | 0 |
| 58 | 1 | 0 | 0 | 0 | 0 |
| 64 | 1 | 0 | 0 | 0 | 0 |
| 59 | 1 | 50 | 0 | 0 | 0 |
| 56 | 1 | 0 | 0 | 0 | 0 |
| 62 | 1 | 0 | 0 | 0 | 2 |
| 5288 | 1 | 0 | 0 | 0 | 0 |
| 5293 | 1 | 0 | 0 | 0 | 0 |
| 5339 | 1 | 0 | 0 | 0 | 0 |
| 5233 | 2 | 50 | 0 | 850 | 21 |
| 4519 | 2 | 700 | 500 | 500 | 21 |
| 41 | 2 | 2250 | 700 | 1450 | 1 |
| 61 | 2 | 400 | 50 | 450 | 6 |
| 63 | 2 | 3200 | 150 | 1650 | 5 |
| 57 | 2 | 150 | 0 | 1150 | 20 |
| 5264 | 2 | 2450 | 400 | 1850 | 2 |
| 53 | 2 | 1750 | 900 | 1200 | 0 |
| 60 | 2 | 500 | 0 | 1150 | 32 |

* Group 1 = Treated; Group 2 = Control
